# Supplementary material for: Resolving the full spectrum of human genome variation using Linked-Reads
Source: Genome Res. 2019 Apr;29(4):635–45. doi: 10.1101/gr.234443.118 (PMC6442396; doi:10.1101/gr.234443.118)
Supplement: Supplemental Material [file supp_gr.234443.118_Supplemental_longranger-2.2.zip › longranger-2.2.tar.bz2!/lr2.2-public/lib/stan/cmdstan/stan/doc/api/html/classstan_1_1mcmc_1_1adapt__diag__e__static__hmc.html]

Stan: stan::mcmc::adapt\_diag\_e\_static\_hmc< M, BaseRNG > Class Template Reference


|  |  |
| --- | --- |
|  | Stan  2.5.0  probability, sampling & optimization |


- Main Page
- Related Pages
- Namespaces
- Classes
- Files

- Class List
- Class Index
- Class Hierarchy
- Class Members

All Classes Namespaces Files Functions Variables Typedefs Enumerations Enumerator Friends Macros Pages

- stan
- mcmc
- adapt\_diag\_e\_static\_hmc

Public Member Functions |
List of all members

stan::mcmc::adapt\_diag\_e\_static\_hmc< M, BaseRNG > Class Template Reference

`#include <adapt_diag_e_static_hmc.hpp>`

Inheritance diagram for stan::mcmc::adapt\_diag\_e\_static\_hmc< M, BaseRNG >:

|  |  |
| --- | --- |
| Public Member Functions | |
|  | adapt\_diag\_e\_static\_hmc (M &m, BaseRNG &rng, std::ostream \*o=&std::cout, std::ostream \*e=0) |
|  | |
|  | ~adapt\_diag\_e\_static\_hmc () |
|  | |
| sample | transition (sample &init\_sample) |
|  | |
| void | disengage\_adaptation () |
|  | |
| Public Member Functions inherited from stan::mcmc::diag\_e\_static\_hmc< M, BaseRNG > | |
|  | diag\_e\_static\_hmc (M &m, BaseRNG &rng, std::ostream \*o=&std::cout, std::ostream \*e=0) |
|  | |
| Public Member Functions inherited from stan::mcmc::base\_static\_hmc< M, diag\_e\_point, diag\_e\_metric, expl\_leapfrog, BaseRNG > | |
|  | base\_static\_hmc (M &m, BaseRNG &rng, std::ostream \*o, std::ostream \*e) |
|  | |
|  | ~base\_static\_hmc () |
|  | |
| sample | transition (sample &init\_sample) |
|  | |
| void | write\_sampler\_param\_names (std::ostream &o) |
|  | |
| void | write\_sampler\_params (std::ostream &o) |
|  | |
| void | get\_sampler\_param\_names (std::vector< std::string > &names) |
|  | |
| void | get\_sampler\_params (std::vector< double > &values) |
|  | |
| void | set\_nominal\_stepsize\_and\_T (const double e, const double t) |
|  | |
| void | set\_nominal\_stepsize\_and\_L (const double e, const int l) |
|  | |
| void | set\_T (const double t) |
|  | |
| void | set\_nominal\_stepsize (const double e) |
|  | |
| double | get\_T () |
|  | |
| int | get\_L () |
|  | |
| Public Member Functions inherited from stan::mcmc::base\_hmc< M, diag\_e\_point, diag\_e\_metric, expl\_leapfrog, BaseRNG > | |
|  | base\_hmc (M &m, BaseRNG &rng, std::ostream \*o, std::ostream \*e) |
|  | |
| void | write\_sampler\_state (std::ostream \*o) |
|  | |
| void | get\_sampler\_diagnostic\_names (std::vector< std::string > &model\_names, std::vector< std::string > &names) |
|  | |
| void | get\_sampler\_diagnostics (std::vector< double > &values) |
|  | |
| void | seed (const Eigen::VectorXd &q) |
|  | |
| void | init\_stepsize () |
|  | |
| diag\_e\_point & | z () |
|  | |
| double | get\_nominal\_stepsize () |
|  | |
| double | get\_current\_stepsize () |
|  | |
| virtual void | set\_stepsize\_jitter (const double j) |
|  | |
| double | get\_stepsize\_jitter () |
|  | |
| void | sample\_stepsize () |
|  | |
| Public Member Functions inherited from stan::mcmc::base\_mcmc | |
|  | base\_mcmc (std::ostream \*o, std::ostream \*e) |
|  | |
| virtual | ~base\_mcmc () |
|  | |
| std::string | name () |
|  | |
| Public Member Functions inherited from stan::mcmc::stepsize\_var\_adapter | |
|  | stepsize\_var\_adapter (int n) |
|  | |
| stepsize\_adaptation & | get\_stepsize\_adaptation () |
|  | |
| var\_adaptation & | get\_var\_adaptation () |
|  | |
| void | set\_window\_params (unsigned int num\_warmup, unsigned int init\_buffer, unsigned int term\_buffer, unsigned int base\_window, std::ostream \*e=0) |
|  | |
| Public Member Functions inherited from stan::mcmc::base\_adapter | |
|  | base\_adapter () |
|  | |
| virtual void | engage\_adaptation () |
|  | |
| bool | adapting () |
|  | |

|  |  |
| --- | --- |
| Additional Inherited Members | |
| Protected Member Functions inherited from stan::mcmc::base\_static\_hmc< M, diag\_e\_point, diag\_e\_metric, expl\_leapfrog, BaseRNG > | |
| void | update\_L\_ () |
|  | |
| Protected Attributes inherited from stan::mcmc::base\_static\_hmc< M, diag\_e\_point, diag\_e\_metric, expl\_leapfrog, BaseRNG > | |
| double | T\_ |
|  | |
| int | L\_ |
|  | |
| Protected Attributes inherited from stan::mcmc::base\_hmc< M, diag\_e\_point, diag\_e\_metric, expl\_leapfrog, BaseRNG > | |
| diag\_e\_point | z\_ |
|  | |
| expl\_leapfrog< diag\_e\_metric  < M, BaseRNG >, diag\_e\_point > | integrator\_ |
|  | |
| diag\_e\_metric< M, BaseRNG > | hamiltonian\_ |
|  | |
| BaseRNG & | rand\_int\_ |
|  | |
| boost::uniform\_01< BaseRNG & > | rand\_uniform\_ |
|  | |
| double | nom\_epsilon\_ |
|  | |
| double | epsilon\_ |
|  | |
| double | epsilon\_jitter\_ |
|  | |
| Protected Attributes inherited from stan::mcmc::base\_mcmc | |
| std::string | name\_ |
|  | |
| std::ostream \* | out\_stream\_ |
|  | |
| std::ostream \* | err\_stream\_ |
|  | |
| Protected Attributes inherited from stan::mcmc::stepsize\_var\_adapter | |
| stepsize\_adaptation | stepsize\_adaptation\_ |
|  | |
| var\_adaptation | var\_adaptation\_ |
|  | |
| Protected Attributes inherited from stan::mcmc::base\_adapter | |
| bool | adapt\_flag\_ |
|  | |

## Detailed Description

### template<typename M, class BaseRNG> class stan::mcmc::adapt\_diag\_e\_static\_hmc< M, BaseRNG >

Definition at line 17 of file adapt\_diag\_e\_static\_hmc.hpp.

## Constructor & Destructor Documentation

template<typename M , class BaseRNG >

|  |  |  |  |
| --- | --- | --- | --- |
| stan::mcmc::adapt\_diag\_e\_static\_hmc< M, BaseRNG >::adapt\_diag\_e\_static\_hmc | ( | M & | *m*, |
|  |  | BaseRNG & | *rng*, |
|  |  | std::ostream \* | *o* = `&std::cout`, |
|  |  | std::ostream \* | *e* = `0` |
|  | ) |  |  |

inline

Definition at line 22 of file adapt\_diag\_e\_static\_hmc.hpp.

template<typename M , class BaseRNG >

|  |  |  |  |  |
| --- | --- | --- | --- | --- |
| stan::mcmc::adapt\_diag\_e\_static\_hmc< M, BaseRNG >::~adapt\_diag\_e\_static\_hmc | ( |  | ) |  |

inline

Definition at line 28 of file adapt\_diag\_e\_static\_hmc.hpp.

## Member Function Documentation

template<typename M , class BaseRNG >

|  |  |  |  |  |
| --- | --- | --- | --- | --- |
| void stan::mcmc::adapt\_diag\_e\_static\_hmc< M, BaseRNG >::disengage\_adaptation | ( |  | ) |  |

inlinevirtual

Reimplemented from stan::mcmc::base\_adapter.

Definition at line 55 of file adapt\_diag\_e\_static\_hmc.hpp.

template<typename M , class BaseRNG >

|  |  |  |  |  |  |
| --- | --- | --- | --- | --- | --- |
| sample stan::mcmc::adapt\_diag\_e\_static\_hmc< M, BaseRNG >::transition | ( | sample & | *init\_sample* | ) |  |

inlinevirtual

Implements stan::mcmc::base\_mcmc.

Definition at line 30 of file adapt\_diag\_e\_static\_hmc.hpp.

---

The documentation for this class was generated from the following file:

- src/stan/mcmc/hmc/static/adapt\_diag\_e\_static\_hmc.hpp


---

[ Stan Home Page ]

*© 2011–2014,
Stan Development Team.*
